# Signaling through retinoic acid receptors is essential for mammalian uterine receptivity and decidualization

**DOI:** 10.1172/jci.insight.150254

**Published:** 2021-09-08

**Authors:** Yan Yin, Meade E. Haller, Sangappa B. Chadchan, Ramakrishna Kommagani, Liang Ma

**Affiliations:** 1 Division of Dermatology, Department of Medicine,; 2 Center for Reproductive Health Sciences, and; 3 Department of Obstetrics and Gynecology, Washington University School of Medicine, St. Louis, Missouri, USA.

**Keywords:** Reproductive Biology, Fertility

## Abstract

Retinoic acid (RA) signaling has long been speculated to regulate embryo implantation, because many enzymes and proteins responsible for maintaining RA homeostasis and transducing RA signals are tightly regulated in the endometrium during this critical period. However, due to a lack of genetic data, it was unclear whether RA signaling is truly required for implantation and which specific RA signaling cascades are at play. Herein we utilize a genetic murine model that expresses a dominant-negative form of RA receptor (RAR) specifically in female reproductive organs to show that functional RA signaling is fundamental to female fertility, particularly implantation and decidualization. Reduction in RA signaling activity severely affects the ability of the uterus to achieve receptive status and decidualize, partially through dampening follistatin expression and downstream activin B/bone morphogenetic protein 2 signaling. To confirm translational relevance of these findings to humans, human endometrial stromal cells (hESCs) were treated with a pan-RAR antagonist to show that in vitro decidualization is impaired. RNA interference perturbation of individual *RAR* transcripts in hESCs revealed that RARα in particular was essential for proper decidualization. These data provide direct functional evidence that uterine RAR-mediated RA signaling was crucial for mammalian embryo implantation, and its disruption led to failure of uterine receptivity and decidualization, resulting in severely compromised fertility.

## Introduction

During early pregnancy, the mammalian endometrium responds to changing ovarian hormones and signaling cues from embryos. The endometrium undergoes extensive growth and differentiation to become appropriately receptive to the incoming blastocysts for implantation (1, 2). Commonly referred to as the “window of implantation,” these complex endometrial events must happen in rapid succession in a short time frame in order to achieve a receptive phase. In the mouse, the implantation window starts the morning of 3.5 days post coitum (dpc; 0.5 dpc = 1200 hours of the day of vaginal plug), when fertilized eggs complete their development through the blastocyst stage and descend into the uterus. By this time, the receiving luminal epithelium (LE) ceases proliferation and initiates differentiation under the influence of rising progesterone (P4) and a small estradiol (E2) surge, whereas the underlying stromal cells undergo extensive proliferation and start to differentiate into morphologically and functionally distinct decidual cells. Toward the evening of 4.5 dpc, the implantation window closes and the uterus enters the refractory phase, during which time the embryos cannot implant. The rapid uterine changes that define the implantation window are tightly regulated by a network of signaling pathways orchestrated by hormones, growth factors, cytokines, and transcription factors. The importance of uterine receptivity genes is evidenced by the severe implantation and/or decidualization defects that occur in response to loss-of-function mutations (3–10).

Retinoic acid (RA), a physiologically active metabolite of its inactive precursor retinol (vitamin A), is essential for many biological processes, including cell survival, differentiation, and apoptosis (11). Vitamin A deficiency, as well as genetically disrupting RA function, leads to defects in the development of many organs and tissues, including the nervous system, kidney, skeleton, heart, lung, and urogenital tracts (12). RA exerts its biological functions mainly through binding to the nuclear RA receptors (RARs) facilitated by the cellular retinoic acid–binding proteins (CRABPs) or, less frequently, to the noncanonical peroxisome proliferator–activated receptor β/γ (PPARβ/γ) facilitated by fatty acid–binding protein 5 (FABP5). The ratio of intracellular lipid-binding proteins CRABP2 to FABP5 tips the balance toward 1 signaling pathway or the other, which frequently has opposing effects (13). Both RARs and PPARs form heterodimers with retinoid X receptors (RXRs), and they regulate target gene expression by directly binding to RA response elements (RAREs) and peroxisome proliferator response elements, respectively. In addition to these 2 signaling cascades, RA can also bind to cytoplasmic RARs and trigger rapid kinase phosphorylation, which in turn regulates downstream signaling events (14).

Previous studies have implicated RA signaling in regulating female fertility. In both the rodent and human endometrium, expressions of RA-synthesizing (aldehyde dehydrogenase and retinaldehyde dehydrogenase) and -metabolizing enzymes (CYP26), as well as RA-binding proteins that regulate its bioavailability (CRBPs/CRABPs/FABP5), are temporally and spatially controlled during early pregnancy (15–17), suggesting their involvement in uterine receptivity and embryo implantation. In addition, high expression of RA signaling receptors, including RARs, PPARβ/γ, and RXRs, has been reported at implantation sites of human and rodent endometrium (17–19). In cultured human endometrial stromal cells (hESCs), gene silencing of CRABP2 and FABP5 by siRNA inhibits and promotes decidualization, respectively (20), suggesting that RA/RAR signaling favors decidualization. In seemingly contradictory in vitro data, treatment of RA at pharmacological levels in hESC culture appears to impair decidualization (17, 21). Given the complex genomic and nongenomic downstream events elicited by RA in various tissues and the absence of any uterine data from genetic animal models, the definitive role of RA signaling in implantation requires clarification.

In the current study, we utilized a previously characterized mouse strain that carries the *RaraT403* truncated form of human *RARα* knocked in to the *Rosa26* locus to dissect the role of RA/RAR signaling in embryo implantation (22). Cre-mediated recombination removes the floxed-STOP sequence upstream of the *RaraT403*, resulting in expression of the dominant-negative form of RARα (hereinafter referred to as *Rara*DN), and subsequent inhibition of endogenous RAR-mediated transcriptional regulation of target genes. We showed that functional RAR signaling is required for mammalian uterine receptivity and decidualization both in the mouse model and in cultured hESCs.

## Results

### RaraDN^Pgr^ females are severely subfertile.

**Figure 1:**
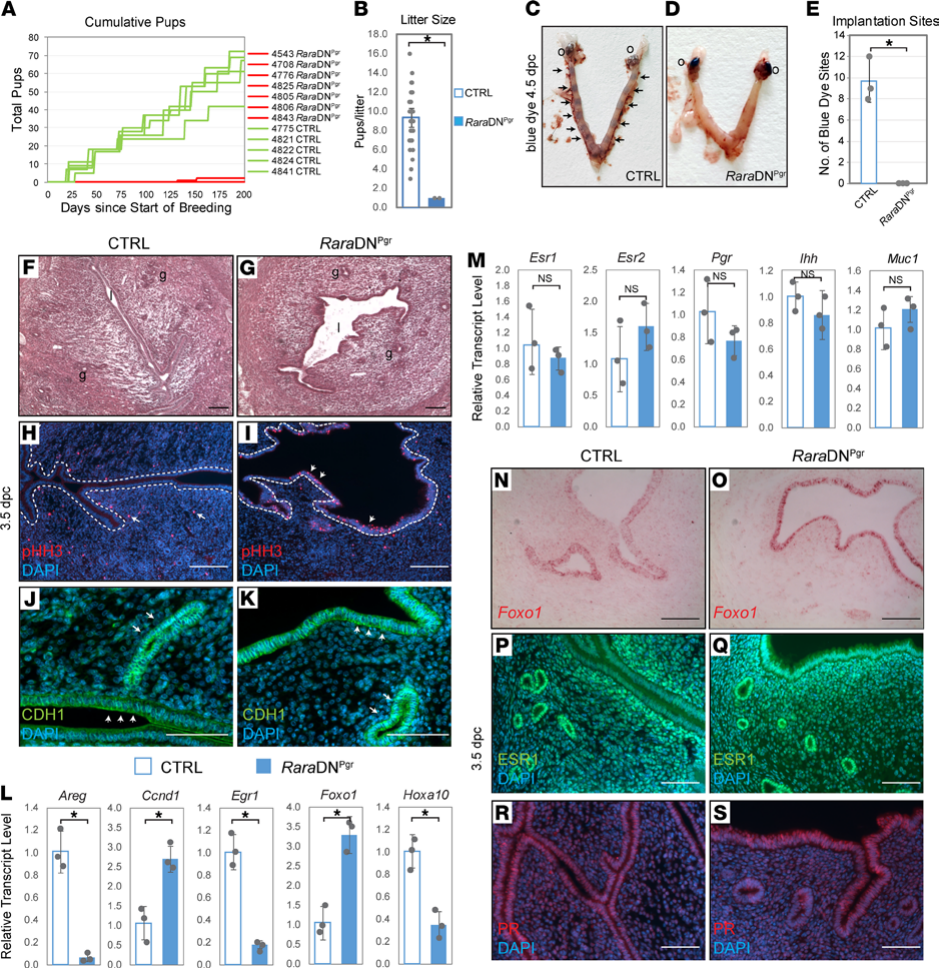
Impaired female fertility due to implantation failure in *Rara*DN^Pgr^ mice. (**A**) Cumulative number of pups produced by individual CTRL (green) and *Rara*DN^Pgr^ females (red) over the period of 200 days. (**B**) Average number of pups per litter produced by the females presented in **A**. (**C** and **D**) Representative images of visualization of implantation sites (arrows) by Blue Dye injection on 4.5 dpc. (**E**) Quantification of implantation sites from CTRL (9.7 ± 2.1, *n* = 3) and *Rara*DN^Pgr^ females (0.0 ± 0.0, *n* = 3, *P* = 0.0013). (**F** and **G**) H&E staining of *Rara*DN^Pgr^ and CTRL uteri on 3.5 dpc. (**H** and **I**) Immunofluorescence detecting pHH3-positive proliferating cells on 3.5 dpc (arrowheads, uterine epithelial cells; arrows, proliferating stromal cells; dashed lines outline the luminal epithelia). (**J** and **K**) CDH1 immunofluorescence on 3.5 dpc uterine sections. Note in the CTRL, reduced CDH1 level is obvious in the luminal epithelium (**J**, arrowheads) when compared with glandular epithelium (**J**, arrows), whereas this difference is negligible in *Rara*DN^Pgr^ uterus (**K**). (**L** and **M**) Gene expression on 3.5 dpc determined by qRT-PCR, normalized to levels of housekeeping gene *Rpl7* and the average transcript level of CTRL samples was set to 1. (**N** and **O**) RNAscope in situ hybridization of *Foxo1* showing elevated transcript level in the mutant epithelium. (**P**–**S**) IF staining of ESR1 (**P** and **Q**) and PR (**R** and **S**) revealed no apparent differences in expression. *P < 0.05; ns. Scale bars: 50 μm. CTRL, controls; dpc, days post coitum; O, ovaries; g, glands; l, lumen; IF, immunofluorescence; pHH3, phospho-histone H3; ESR1, estrogen receptor 1; PR, progesterone receptor.

To generate mice with disrupted RA signaling in the female reproductive tracts, *Rara*DN^fl/+^ mice were mated to *Pgr*-Cre mice, resulting in offspring females carrying both alleles (*Rara*DN^fl/+^; *Pgr*-Cre, hereafter referred to as *Rara*DN^Pgr^) and littermate controls (CTRLs; no *Pgr*-Cre). *Pgr*-Cre–mediated gene recombination in the uterus is first detected in the luminal and glandular epithelia starting at 2 weeks of age, and gradually expands to the stroma and myometrium (23). To assess disruption of RAR signaling, we examined the expression of several *Hox* genes, known direct downstream targets of RAR, in the uteri of ovariectomized CTRL and *Rara*DN^Pgr^ mice (Supplemental Figure 1; supplemental material available online with this article; https://doi.org/10.1172/jci.insight.150254DS1). Reduction in expression of the majority of the *Hox* genes in the *Rara*DN^Pgr^ uteri demonstrates that RAR signaling was successfully disrupted in these animals. Female fertility was evaluated by breeding test of WT males with either *Rara*DN^Pgr^ or CTRL females and tracking the number of litters and pups produced by each female for 200 consecutive days. Of the *Rara*DN^Pgr^ females tested, 6 of 7 were completely sterile, producing 0 litters over the span of 7 months (Figure 1A). The remaining *Rara*DN^Pgr^ female produced only 2 litters, each consisting of only 1 pup after a long hiatus after mating setup (Figure 1, A and B). *Rara*DN^Pgr^ females as a whole are therefore severely subfertile, producing significantly fewer litters and pups than CTRLs (Table 1; *P* = 9.8 × 10^–9^ and 7.2 × 10^–9^, respectively, *n* = 5 for CTRL and *n* = 7 for *Rara*DN^Pgr^). No apparent developmental and behavioral abnormalities were observed in the 2 pups born to the *Rara*DN^Pgr^ sterility escapee. Vaginal plugs were consistently observed in the *Rara*DN^Pgr^ females, ruling out the possibility of behavioral issues preventing mating. As these females never presented palpable pregnancies, early-stage pregnancy defects were suspected.

### RaraDN^Pgr^ females exhibit implantation defects.

Mouse embryo implantation occurs between 3.5 and 4.5 dpc, when blastocysts attach to the LE, which in turn triggers the underlying stromal cells to undergo decidualization. Successful implantation is accompanied by increased local vascular permeability, which can be visualized by tail vein injection of Chicago blue dye. Distinct blue dots indicating implantation sites were easily detectable along the uterine horns of CTRL mice on 4.5 dpc (Figure 1D, arrows, 9.7 ± 2.1, *n* = 3) but were completely absent in the *Rara*DN^Pgr^ uteri (Figure 1, C and E; 0.0 ± 0.0, *n* = 3, *P* = 0.0013).

Many factors, alone or in combination, can contribute to failed pregnancy at an early stage, including abnormal ovulation, irregular ovarian hormone levels, and poor uterine receptivity. Because *Pgr-*Cre is also active in the adult ovary, including corpora lutea and human chorionic gonadotropin–stimulated granulosa cells (23), it is essential to investigate whether disturbance of RA signaling affects ovarian functions. Evaluation of the *Rara*DN^Pgr^ ovaries on 3.5 dpc revealed normal histology with presence of multiple corpus lutea, which are the remnants of successfully matured vesicular follicles after ovulation (Supplemental Figure 2, A and B). Morphologically normal blastocysts were recovered from *Rara*DN^Pgr^ females by flushing the oviducts and uterine horns on 3.5 dpc (Supplemental Figure 2A, inset), and no significant difference was observed in the number of retrieved blastocysts at this stage (Supplemental Figure 2C; CTRL 8.5 ± 3.5, *n* = 3; vs. *Rara*DN^Pgr^ 6.5 ± 2.1, *n* = 3, *P* = 0.27), indicating normal fertilization rates. In addition, evaluation of serum ovarian hormone levels at this stage by ELISA revealed no significant differences (Supplemental Figure 2D). Together these data indicate normal ovarian function in *Rara*DN^Pgr^ females.

### RaraDN^Pgr^ females exhibit uterine receptivity defects.

Successful embryo implantation depends on the achievement of uterine receptivity through a series of molecular, hormonal, and morphological changes. In the 3.5 dpc mouse uterus, the LE typically ceases proliferation under the influence of decreased E2 and P4 levels to prime for rapid remodeling and embryo embedding (24). Meanwhile, luminal epithelial cells turn off genes for apical–basal polarity like Cadherin1 (CDH1, also known as E-cadherin) to allow attachment of trophoblast cells to their apical pole (25). To assess the status of uterine receptivity, we first examined gross uterine morphology at this stage and found no overt abnormalities except for aberrant luminal closure (Figure 1, F and G). Uteri of CTRL 3.5 dpc females exhibited typical signs of receptivity, i.e., halted epithelial proliferation evidenced by limited phospho-histone H3 staining (Figure 1H) and reduced CDH1 expression exclusively in the LE (Figure 1J, arrowheads). By contrast, the *Rara*DN^Pgr^ uteri sustained high LE proliferating activity (Figure 1I, arrowheads) and high CDH1 expression (Figure 1K, arrowheads) in the LE, consistent with a prereceptive or nonreceptive uterus.

Quantitative RT-PCR (qRT-PCR) was performed to further interrogate expression of genes involved in uterine receptivity. Amphiregulin (*Areg*), a member of the epidermal growth factor family, is upregulated exclusively in the uterine epithelium on 3.5 dpc surrounding the embedding embryos in a P4-dependent manner (26). This upregulation was absent in the *Rara*DN^Pgr^ mutant (Figure 1L). Expression of early growth response gene 1 (*Egr1*), a zinc finger transcription factor that is crucial for cell proliferation and angiogenesis, was previously reported to be induced in the subluminal stroma surrounding the blastocysts (27), but it is barely detectable in the mutant uterus on 3.5 dpc. Previous studies in ovarian hormone-responsive cells including uterine epithelial cells have shown that E2 signaling can promote LE cell proliferation by transactivating expression of the cell cycle gene cyclin D1 (*Ccnd1*), as well as facilitating its nuclear translocation (28, 29). *Rara*DN^Pgr^ uteri exhibit a marked increase in *Ccnd1* mRNA, which may contribute to the mutant’s aberrant epithelial proliferation. Deregulation of transcription factors essential for uterine receptivity and embryo implantation was also evident in the *Rara*DN^Pgr^ uteri on 3.5 dpc, including those expressed in the epithelial compartment, such as Forkhead Box O1 (*Foxo1*; ref. 30), and those exclusively expressed in the stroma, such as Homeobox A10 (*Hoxa10*; ref. 31). RNAscope in situ hybridization of *Foxo1* revealed that its elevation in the mutant is confined to the uterine epithelium (Figure 1, N and O). In addition, analysis of a subset of gold standard receptivity biomarkers used in customized endometrial receptivity arrays for clinical endometrial evaluation in humans (32, 33) revealed markedly reduced expression of many receptivity biomarkers in the *Rara*DN^Pgr^ uteri during the periimplantation period (Supplemental Figure 3). Even though many of these genes have been reported to be regulated by ovarian hormones, the changes we observed in the *Rara*DN^Pgr^ mutant are unlikely to be elicited solely by altered hormone signaling, because serum ovarian hormone level (Supplemental Figure 2) as well as uterine expression of ovarian hormone receptors and some of their well-established targets remained unchanged (Figure 1M). Immunofluorescence of estrogen receptor 1 (ESR1) and progesterone receptor (PR) further confirmed that the ovarian hormone receptors were expressed at normal locations and levels in *Rara*DN^Pgr^ females comparable to their WT counterparts (Figure 1, P–S).

### Decidualization is compromised in RaraDN^Pgr^ females.

**Figure 2:**
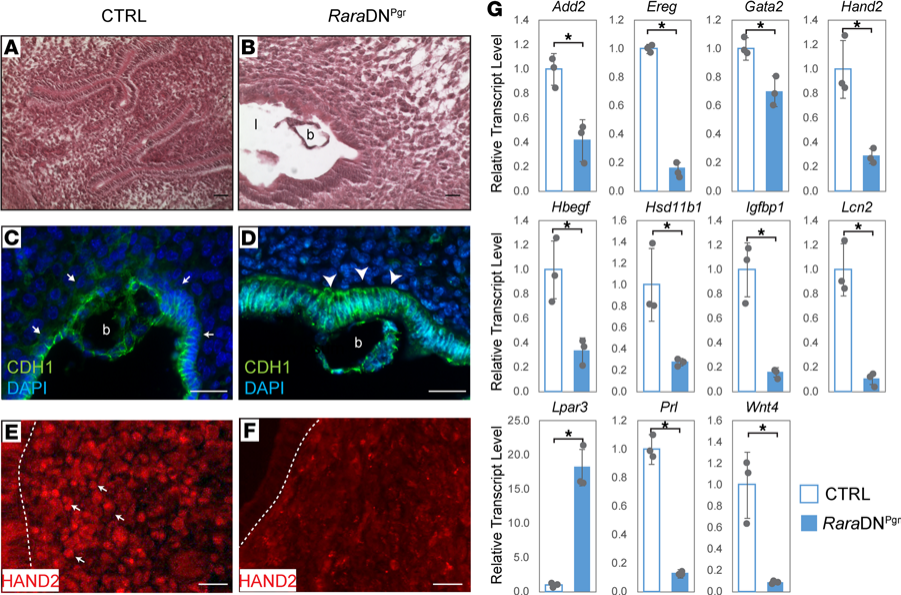
Failure of decidualization in 4.5 dpc *Rara*DN^Pgr^ uterus. (**A** and **B**) H&E staining of *Rara*DN^Pgr^ and CTRL uteri on 4.5 dpc. (**C** and **D**) CDH1 immunofluorescence on 4.5 dpc uterine sections. Note the presence of normal blastocyst in the mutant lumen (**D**), and persistent high CDH1 level in the underlying luminal epithelium, especially at the basal side (arrowheads, compared with CTRL arrows in **C**). (**E** and **F**) Immunofluorescence detection of HAND2 protein in the nuclei of decidual cells in the CTRL (arrows, **E**), which is absent in the mutant (**F**). (**G**) Relative transcript levels of genes involved in decidualization by qRT-PCR. Scale bars: 50 μm. *P < 0.05. dpc, days post coitum; CTRL, control; l, lumen; b, blastocyst; CDH1, cadherin1; HAND2, heart and neural crest derivatives expressed transcript 2.

Despite the absence of luminal closure, which is thought to help immobilize the embryos for implantation, blastocyst attachment appears to successfully occur in the *Rara*DN^Pgr^ uteri on 4.5 dpc (Figure 2, B and D, compared with Figure 2, A and C), raising the possibility that failures in subsequent pregnancy events also contribute to the fertility defects. As we and others have previously reported (34, 35), during embryo attachment, strong CDH1 expression is present only in the apical poles of the uterine epithelium and barely detectable on the basal side (Figure 2C, arrows). Interestingly, this polarized localization of CDH1 is absent in the *Rara*DN^Pgr^ uteri; strong CDH1 staining was observed on both sides (Figure 2D, arrowheads). After embryo attachment, fibroblastic uterine mesenchymal cells undergo decidualization. Decidualization is the rapid proliferation and differentiation of these cells into morphologically distinct decidual cells, which provide a plethora of growth factors and cytokines to support embryo development and serve an immunoregulatory role during early pregnancy. To investigate whether decidualization is affected in the *Rara*DN^Pgr^ females, we first examined the expression of known decidualization markers during natural pregnancy in these mutants. Transcription factor heart and neural crest derivatives expressed transcript 2 (HAND2) plays a critical role in uterine receptivity and decidualization in the mouse, and its expression is induced in endometrial stromal cells starting on 3.5 dpc and increases over time (7, 36). It modulates stromal–epithelial communications through negative regulation of FGF signaling and genetic ablation of *Hand2* in the mouse lead to female infertility largely due to decidualization failure (7, 36). Immunofluorescence revealed that HAND2 protein exhibited nuclear localization in the CTRL subepithelial stromal cells on 4.5 dpc (Figure 2E, arrows), but its level was dramatically reduced and its nuclear localization undetectable in the mutant uterus (Figure 2F). This reduction in *Hand2* levels was confirmed at the transcript level by qRT-PCR using RNA extracted from whole uterine tissues on 4.5 dpc (Figure 2G). Expression of an array of genes involved in decidualization was evaluated by qRT-PCR, and mutant uteri exhibited significant decreases in the majority of them, including *Add2*, *Ereg*, *Gata2*, *Hbegf*, *Hsd11b1*, *Igfbp1*, *Lcn2*, *Prl*, and *Wnt4*. *Lpar3*, a gene encoding lysophosphatidic acid receptor 3, a G protein–coupled receptor for lysophosphatidic acid that fine-tunes the local balance of P4 and E2 signaling during implantation, is highly expressed in the 3.5 dpc uterus and sharply shut down at 4.5 dpc during normal implantation (37, 38). In the mutant 4.5 dpc uterus, *Lpar3* transcript level remained high, consistent with the failed implantation observation. Together these results demonstrate a decidualization failure in *Rara*DN^Pgr^ females in the setting of natural pregnancy.

**Figure 3:**
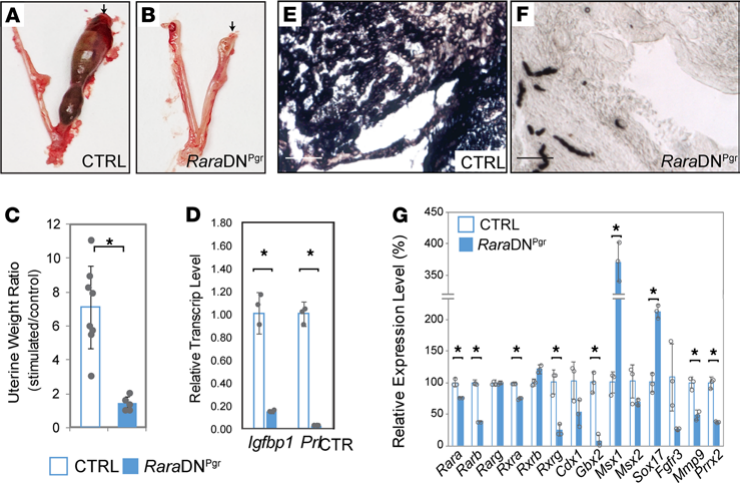
*Rara*DN^Pgr^ mutant uterus does not respond to artificial decidualization stimuli in vivo. (**A** and **B**) Representative images of artificially decidualized uteri 5 days after stimulation. Arrows indicate the uterine horn that received intrauterine oil infusion; contralateral horns serve as controls. (**C**) Uterine weight ratio (wet weight stimulated/wet weight unstimulated) was calculated for each animal, and graphed as mean ± SD (CTRL, 7.1 ± 2.4, *n* = 8; RaraDNPgr, 1.4 ± 2.4, *n* = 5; *P* = 0.00036). (**D**) Gene expression data of decidualization markers, Igfbp1 and Prl, in the stimulated uterine horn by qRT-PCR. (**E** and **F**) AP activity in the stimulated uterine horns visualized by dark color development from AP substrate BCIP/NBT. (**G**) Gene expression analyses of RARs and RAR targets comparing RNA extracted from stimulated CTRL and *Rara*DN^Pgr^ uteri by qRT-PCR. *P < 0.05. Scale bars: 50 μm. CTRL, control; AP, alkaline phosphatase; RA, retinoic acid; RAR, RA receptor.

**Figure 4:**
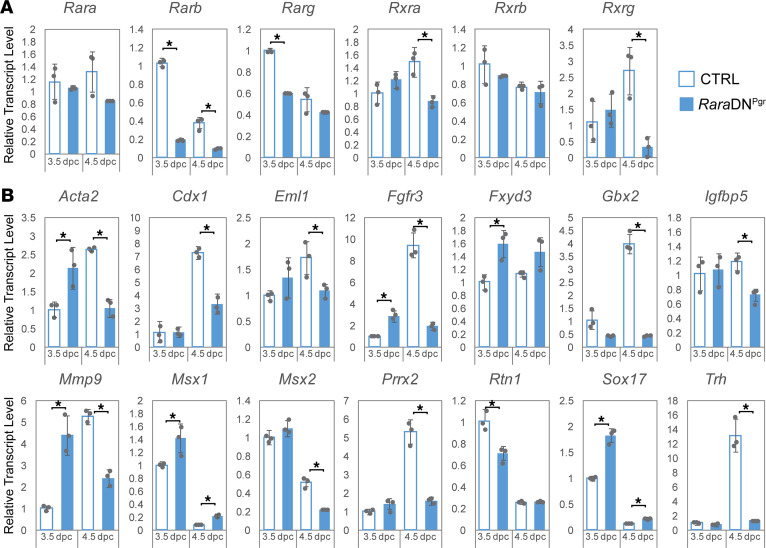
Changes in expression of RAR genes and known RAR targets in the *Rara*DN^Pgr^ uterus during the periimplantation period. Gene expression by qRT-PCR performed on whole uterine RNA extract on 3.5 and 4.5 dpc detecting RARs (**A**) and targets (**B**). *P < 0.05 comparing CTRL uteri of same time point. RAR, retinoic acid receptor; dpc, days post coitum; CTRL, control.

To rule out the potential involvement of defective embryo attachment and/or defective hormone regulation as a cause for decidualization failure, we performed an artificial decidualization assay. In mice, decidualization of uterine stromal cells can be achieved by intraluminal oil injection into the uterine horns of ovariectomized and hormone-primed females followed by additional hormone treatments after induction. As shown in Figure 3, A and B, disruption of RA signaling in *Rara*DN^Pgr^ uteri renders them nonresponsive to decidual stimuli. Uterine weight gain due to stimuli was completely abolished (Figure 3C), and differentiation markers like *Igfbp1*, *Prl*, and alkaline phosphatase (AP) failed to be induced in the mutant uteri (Figure 3, D–F). Genes encoding RARs as well as some known downstream RAR signaling targets showed differential expression in stimulated mutant uteri relative to CTRLs (Figure 3G). Significant reduction in transcript levels were observed in the mutants for RARs *Rara*, *Rarb*, *Rxra*, and *Rxrg*, as well as RA targets *Cdx1*, *Gbx2*, *Mmp9*, and *Prrx2*. On the other hand, stimulated mutant uteri exhibit drastic increases in mRNA levels for transcription factors *Msx1* and *Sox17* relative to CTRL decidua. Expression of *Msx1* was previously reported to sharply decline after embryo attachment to prepare the uterus for implantation by modulating WNT and FGF signaling between the epithelial and stromal compartments (39). In addition, persistent *Msx1* expression was shown to be associated with uterine receptivity defects observed in *Lif*^–/–^ mice (40). *Sox17* also plays critical roles during implantation through modulating the uterine transcriptome (41). Most of the genes assayed including some of the RARs (Figure 4A) and their downstream targets (Figure 4B) displayed the same trend of expression changes during the periimplantation period of natural pregnancy in *Rara*DN^Pgr^ uteri. These findings provide further support that disrupted expression of RAR downstream targets likely contributed to the decidualization defect observed in the *Rara*DN^Pgr^ mutants. We stress, however, that at present we cannot exclude the possibility that the decidualization defect in these mutants was secondary to the observed uterine receptivity defect. Tissue-specific ablation of RA signaling in the implanting uterus is required to address this point.

### Disrupted RAR-signaling leads to reduced follistatin and aberrant activin signaling.

**Figure 5:**
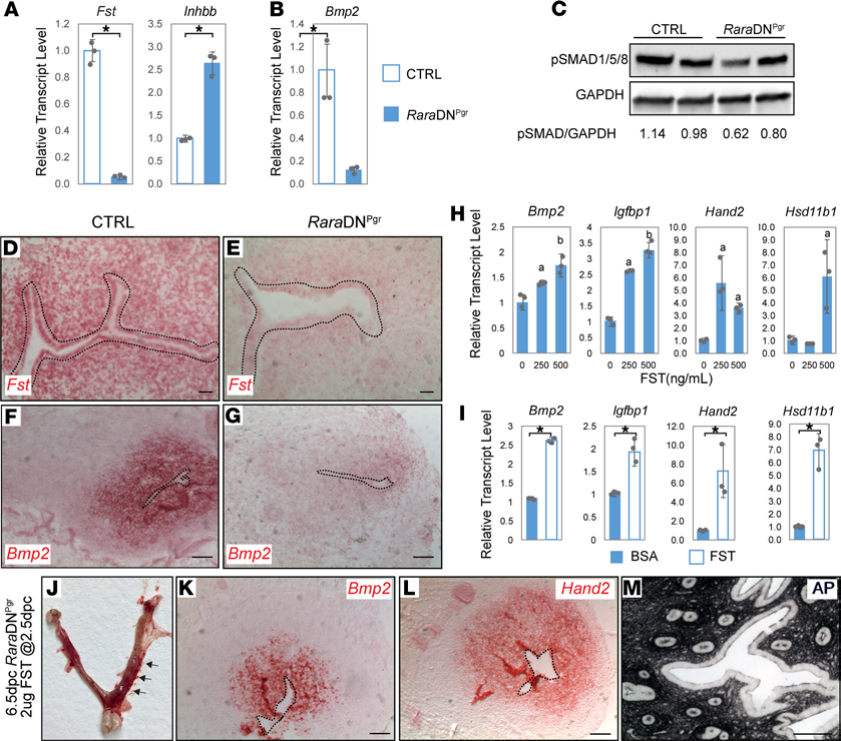
Reduced follistatin expression and downstream changes in activin/BMP signaling are partially responsible for the fertility defects in *Rara*DN^Pgr^ uterus. (**A** and **B**) Relative expression levels of Fst and Inhbb on 3.5 dpc (**A**) and Bmp2 on 4.5 dpc (**B**). (**C**) Western blot for phospho-Smad1/5/8 of whole uteri extract from CTRL and RaraDN^Pgr^ females on 3.5 dpc. Western blot band density was quantified in ImageJ (NIH), and the relative density calculated as the ratio of pSMAD/GAPDH for each sample was listed. (**D**–**G**) RNAscope in situ hybridization of *Fst* on 3.5 dpc (**D** and **E**) and *Bmp2* on 4.5 dpc (**F** and **G**). Positive results manifest as red staining; dotted lines outline the luminal epithelium. (**H**) Gene expression of decidualization markers in isolated endometrial stromal cells from 2.5 dpc *Rara*DN^Pgr^ uteri treated with recombinant mouse FST at indicated concentrations for 48 hours (*n* = 2). (**I**) Gene expression of decidualization markers in uterine segments dissected from 2.5 dpc *Rara*DN^Pgr^ and incubated with luminal agarose beads soaked with BSA or FST for 2 days (*n* = 2). (**J**) Appearance of *Rara*DN^Pgr^ mutant uteri 4 days after receiving 2 μg FST via tail vein injection (arrows point to bulging regions resembling implantation sites). (**K** and **L**) RNAscope in situ hybridization of *Bmp2* (**K**) and *Hand2* (**L**) showed elevated expression in the bulging region. (**M**) BCIP/NBP staining of bulging regions shows extensive alkaline phosphatase activity. Asterisks indicate *P* < 0.05 by *t* test; a indicates *P* < 0.05 by 1-way ANOVA between drug group and control group; and b indicates *P* < 0.05 by 1-way ANOVA between different doses. Scale bars: 50 μm. dpc, days post coitum; CTRL, control; BMP, bone morphogenetic protein 2.

During gene expression analysis, we observed a striking decrease in the expression of follistatin (*Fst*) in the *Rara*DN^Pgr^ on 3.5 dpc (Figure 5A). This is of particular interest because previous studies revealed that *Fst* is a direct transcriptional target of RA signaling, containing RAREs in its promoter region (42). Additionally, *Pgr*-Cre–mediated genetic deletion of *Fst* leads to female fertility defects very similar to our *Rara*DN^Pgr^ mutants (43). Accompanying the sharp reduction of *Fst*, expression of inhibin βb (*Inhbb*), components of activin B and downstream target of FST signaling, was significantly upregulated (Figure 5A). In the uterine-specific *Fst* knockout model, absence of FST and elevated activin B activity caused reduction in BMP signaling, especially BMP2, through the activin/SMAD signaling pathway (43). In line with this notion, we observed a similar reduction in *Bmp2* expression on 4.5 dpc (Figure 5B), as well as reduction in phospho-SMAD1/5/8 (Figure 5C) in *Rara*DN^Pgr^ mutants. RNAscope in situ hybridization was performed, which further confirmed the reduction of *Fst* and *Bmp2* in *Rara*DN^Pgr^ uterus. *Fst* transcript was detected throughout the CTRL uterus on 3.5 dpc (Figure 5D) but was barely detectable in the mutant (Figure 5E). *Bmp2* transcript was detected exclusively in the subepithelial stromal cells in CTRL uterus on 4.5 dpc (Figure 5F), and its expression was markedly reduced in the mutant (Figure 5G). If the fertility defects observed in the *Rara*DN^Pgr^ mice are indeed caused primarily by the loss of *Fst* expression, one would expect the phenotype to be alleviated when FST is supplemented back to the mutant uterus. To test this hypothesis, we isolated uterine stromal cells from 2.5 dpc mutants for in vitro culture and added recombinant mouse FST to the medium at various concentrations. Forty-eight hours after culture, expression of several decidualization markers is elevated by addition of FST; for *Bmp2* and *Igfbp1* these changes are dose-dependent (Figure 5H). Similar restoration of decidual marker expression were observed in a uterine organ culture system where either BSA-soaked or FST-soaked agarose beads were inserted into the lumens of 2.5 dpc *Rara*DN^Pgr^ uterine segments and allowed to culture in vitro for 2 days (Figure 5I). To test whether FST is sufficient to rescue the mutant implantation defects in vivo, FST was administered systemically into 2.5 dpc *Rara*DN^Pgr^ females via tail vein injection. On 6.5 dpc, bulging regions along the uterine horns resembling implantation sites were observed in these mutants (Figure 5J), although they appeared smaller than normal implantation sites at this developmental stage. Sections through the bulging regions revealed elevated *Bmp2* and *Hand2* transcripts (Figure 5, K and L, respectively), as well as extensive AP activity (Figure 5M), indicating restored decidualization in the mutant by FST administration. However, histological analyses did not reveal any uterine closure or embryo presence, suggesting that other aspects of implantation, most likely uterine receptivity, could not be rescued by FST alone. This partial rescue was observed in 2 of 3 *Rara*DN^Pgr^ females tested, with 2 and 3 bulging sites in each animal, respectively. These data together demonstrate that RARs regulated uterine decidualization mainly through FST.

### RAR signaling is essential for decidualization in hESCs.

**Figure 6:**
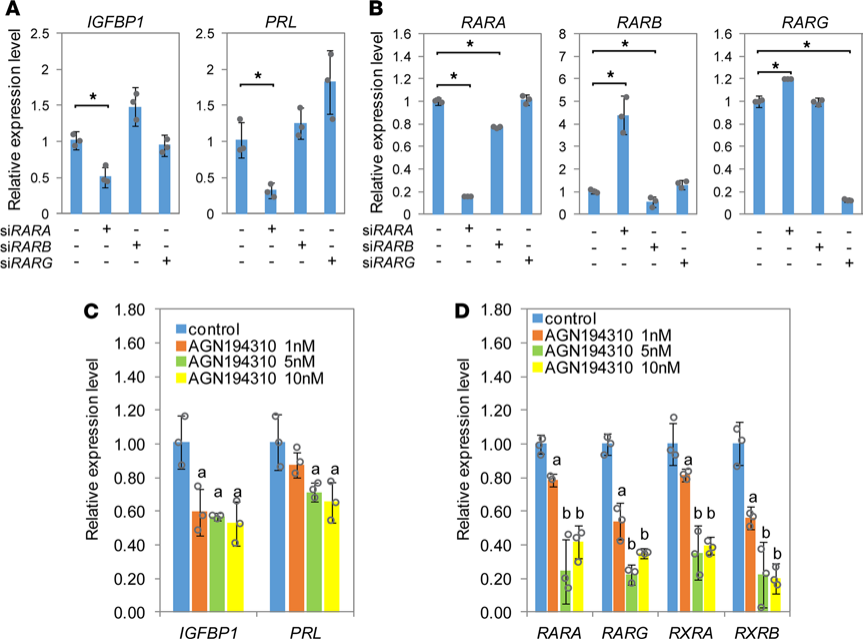
RAR signaling is essential for decidualization of hESCs. (**A** and **B**) Gene expression by qRT-PCR of human decidualization markers (**A**) and RAR genes (**B**) when individual RAR genes are silenced by siRNA. (**C** and **D**) Expression of decidualization markers (**C**) and selective RAR and RXR genes (**D**) when hESCs are treated with pan-RAR antagonist AGN194310 at indicated concentrations. Asterisks indicate *P* < 0.05 by *t* test; a indicates *P* < 0.05 by 1-way ANOVA between drug group and control group; and b indicates *P* < 0.05 by 1-way ANOVA between different doses.

In our previous study, we engineered a fluorescent reporter hESC line and performed genome-wide siRNA screening to identify genes required for normal decidualization (44). A total of 136 genes involved in the RA pathway were among the hits, including 29 upstream and 107 downstream of RAR signaling (Supplemental Figure 4). To investigate the role of RAR signaling in human endometrium, we performed individual siRNA knockdown against the 3 human *RAR* genes (*RARA*, *RARB*, and *RARG*) in hESCs. In particular, knocking down *RARA* significantly inhibited in vitro decidualization of the hESCs, evidenced by a decreased expression of decidualization markers *IGFBP1* and *PRL* (Figure 6A). Successful knockdown of individual *RAR* genes was confirmed by qRT-PCR (Figure 6B). Interestingly, siRNA against *RARA* not only reduced *RARA* expression by more than 80% but also simultaneously resulted in significant increases in *RARB* and *RARG* expression, likely due to a compensatory signaling feedback loop (Figure 6B). By contrast, knocking down *RARB* or *RARG* did not affect hESC decidualization, nor did it elicit significant changes in the expression of other *RAR* genes (Figure 6, A and B).

To further dissect the involvement of RAR genes in implantation, we evaluated the expression of RAR genes by qRT-PCR in both mouse uterus during the periimplantation period and decidualized hESCs. As shown in Supplemental Figure 5, in both model systems, *Rara/RARA* and *Rarg/RARG* were the most abundant isotypes among the *Rar/RAR* genes, whereas *Rxra/RXRA* and *Rxrb/RXRB* were the predominantly expressed *Rxr/RXR* genes. Hormonal regulation of the receptor genes was also examined in cultured hESCs (Supplemental Figure 6). Twenty-four hours of exposure to medroxyprogesterone acetate (MPA) elevated *RARA* transcript level, and this effect was augmented by cotreatment of E2 plus MPA, even though E2 alone did not elicit any changes. *RXRB*, on the other hand, was induced and suppressed by E2 and MPA, respectively, and cotreatment appears to have counteracted each other and canceled out the effect. The expression levels of the other *RAR* and *RXR* genes were not affected by hormone treatment within this time frame.

To further demonstrate dependency of human decidualization on RA signaling, hESCs were treated with a pan-RAR antagonist, AGN194310 (45), at increasing concentrations. As shown in Figure 6C, both decidualization markers *IGFBP1* and *PRL* exhibited a dose-dependent decrease in expression upon drug treatment. The expression of endogenous *RAR* genes, including *RARA*, *RARG*, *RXRA*, and *RXRB*, also exhibited dose-dependent reductions in response to AGN194310 (Figure 6D). Taken together, these results strongly support the notion that RAR signaling, particularly through RARA, was required for in vitro decidualization of hESCs.

## Discussion

In the current study, we generated and characterized a mouse model with conditional disruption of RA/RAR signaling specifically in female reproductive organs. The dominant-negative *Rara*DN allele used in this study has been previously shown to block endogenous RAR-dependent signaling through competitively binding to RAREs (22). The vast majority of females carrying only 1 copy of the *Rara*DN allele in *Pgr*-cre–expressing cells are sterile, whereas 1 is severely subfertile, due to defective uterine receptivity and decidualization. Given that the dominant negative receptor blocks RAR signaling in a dose-dependent manner, and that having 2 alleles of *Rara*DN completely abolishes endogenous RA signaling (22), we expect the detrimental effects on female fertility would be more severe in *Pgr*-cre *Rara*DN*^fl/fl^* females. Our findings also indicate that RA signaling through PPARβ/γ and/or nongenomic pathways could not compensate for the loss of RAR signaling during implantation.

Even though *Pgr*-cre also mediates *Rara*DN expression in the ovary, there is no indication that the mutant ovaries are affected. Not only do ovarian hormone levels remain unchanged in these mutants, but ovulation and fertilization also occur normally. Consistent with our data, genetic ablation of all 3 *Rar* genes as well as that of all 3 RA synthesis enzymes (*Aldh1a1*–*3*) in the developing mouse ovary does not affect ovary differentiation or ovarian function (46). RAR signaling endogenous to the embryos also does not appear to be required for uterine receptivity or decidualization, as transgenic embryos carrying the *Rara*DN allele driven by an SV40 early promoter implant and develop to term when transferred into WT recipient dams (47).

In the absence of ligand, RAR/RXR heterodimers can actively repress target genes by occupying RAREs and complexing with corepressor proteins, such as nuclear receptor corepressor (NCoR) and silencing mediator of retinoic acid (SMRT), to prevent transcription (48–50). The presence of RA induces conformational changes in the ligand-binding domain of RARs, resulting in simultaneous attenuation of affinity for corepressors and increased affinity for coactivators, including histone acetyltransferases and DRIP/TRAP/ARC coactivators and other mediator-containing complexes, to decompress chromatin and transactivate target genes (51–53). Rapid repression of target genes upon RA signaling activation has also been reported extensively (54–56); however, the molecular mechanism is less studied. It is believed that liganded heterodimers recruit polycomb repressive complex 2, histone deacetylase, and coregulator(s) to actively inhibit target gene transcription, but the identity of the coregulator(s) remains unknown. Even though the 3 RAR genes share extensive homology, and in many cases function redundantly, unliganded heterodimers RXR/RARβ and RXR/RARγ interact with SMRT corepressors differently from unliganded heterodimers of RXR/RARα by mediating a substantial level of transactivation rather than repression (57). The *Rara*DN mutant receptor used in this study lacks the carboxyl terminal sequence of the human *RARA* gene but is also highly efficient at inhibiting the other 2 receptors (47). Dose-dependent blocking of transcription activation by this receptor has been demonstrated in various RARE-reporters both in vitro and in vivo (22, 47, 58); however, little is known about its impact on relieving repression or active inhibition of target genes. In the current study, we identified genes that are activated or repressed during the periimplantation window in *Rara*DN^Pgr^ uterus, suggesting both instructive and permissive roles of RAR signaling. Whether these genes were direct transcriptional targets of RAR signaling or their expression reflected a manifestation of changes in a cohort of “master RAR targets” demands further investigation.

In this study, we report that the receptivity and decidualization defects in the *Rara*DN^Pgr^ uterus were partially caused by loss of *Fst* expression. FST, also known as activin-binding protein, is a glycoprotein that regulates TGF-β superfamily signaling, primarily through binding to activin (59). Activin B, homodimer of Inhibin βB, binds to and activates ACVR2A/B and ALK4/7 and in turn phosphorylates SMAD2/3. *Fst* is upregulated during periimplantation in the mouse uterus, which is believed to sequester activin B in order to allow BMP signaling activation (43). Genetic ablation of *Fst* leads to severe female subfertility in mice with receptivity and decidualization defects similar to *Rara*DN^Pgr^ mice (43), and aberrant expression of FST and activins are associated with poor pregnancy outcome in patients undergoing in vitro fertilization (IVF; ref. 60). In *Rara*DN^Pgr^ mice, greatly reduced *Fst* expression was accompanied by increased *Inhbb* expression on 3.5 dpc and loss of *Bmp2* induction on 4.5 dpc. Interestingly, the loss of *Bmp2* and deregulation of other decidualization markers were partially rescued by supplementation of FST protein in isolated mutant endometrial stromal cells, in organ culture, as well as in vivo, suggesting that loss of FST can largely account for the severity of decidualization defects in *Rara*DN^Pgr^ mice. However, *Fst* downregulation is unlikely to be the sole reason for the mutant impaired fertility for 3 reasons. First, gene expression changes in the *Rara*DN^Pgr^ mutants including a wide array of known RA targets were evident that have no known link to the FST and activin signaling pathway. Second, the fertility defects, especially in terms of decidualization, are much more severe in the *Rara*DN^Pgr^ mutants than in the *Fst*-cKO mice. Finally, not all decidualization genes tested were rescued by FST supplementation, e.g., *Prl*. Thus, although FST is an important downstream component of decidual RAR signaling, and although it is likely a direct RAR transcriptional target (42), our findings suggest that a wider network of signaling pathways was at play. Interestingly, the regulation of BMPs by RAR signaling has been reported in other cellular contexts. In the mouse testicular embryonal carcinoma cell line, RA induces *Bmp2* while simultaneously repressing *Bmp4*, specifically through RARα and RARγ (61). In primary bone marrow stromal cultures, the addition of retinaldehyde stimulates *Bmp2* expression, and this induction is dampened by cotreatment of RAR antagonist AGN193109 (62). Whether the regulation of BMPs by RAR signaling is also mediated by FST in these specific cell types is not clear.

In addition to the mouse data, we also demonstrated the requirement of RAR signaling, specifically through RARα, in hESC decidualization. Knocking down *RARA* in hESCs resulted in substantial downregulation of decidualization markers, as well as the elevation of *RARB/G*, possibly by a compensatory mechanism in response to a loss of RARα signaling. Knockdown of *RARB/G*, on the other hand, had no detectable effects on human in vitro decidualization. Treatment of a pan-RAR antagonist also caused a dose-dependent reduction of decidualization marker expression, as well as the expression of major *RAR/RXR* genes. Infertility due to vitamin A deficiency has been reported in humans, and fertility was restored after carefully titrated supplementation of vitamin A back to normal levels (63). A review of the vitamin A content of the top 25 best-selling prenatal vitamins at the United States’ top-grossing online store (Amazon.com) revealed that the percent daily value for pregnant and nursing individuals ranges from 0% to 185% from a variety of precursors, with the best seller having no vitamin A (Supplemental Table 1). Extreme excess maternal vitamin A is a documented teratogen, although this is shown to be largely from feedback inhibition of native RA production in developing embryos (64). Low maternal vitamin A intake can likewise cause birth defects such as diaphragmatic hernia (65). The current study adds to the existing body of data to emphasize that not only is it essential for maternal/fetal health to have biologically appropriate levels of maternal RA, but it is also crucial for uterine receptivity and decidualization to have proper RAR signaling, as shown herein in mice in vivo and in hESCs in vitro.

## Methods

### Mice.

Generation of mice carrying *Rara*DN preceded by a floxed transcriptional/translational STOP sequence was previously described (22), and cryopreserved sperms from mutant mice were provided by Benjamin D. Humphreys in the Division of Nephrology, Washington University School of Medicine. Live mice carrying the *Rara*DN mutation were rederived via IVF at the Mouse Genetics Core at Washington University. Pgr-Cre line was provided by Francesco DeMayo (National Institute of Environmental Health Sciences, Durham, North Carolina, USA; ref. 23) and mated to *Rara*DN^fl/+^ mice to generate offspring carrying both alleles (hereinafter referred to as *Rara*DN^Pgr^) and littermate CTRLs (*Rara*DN^fl/+^). Artificial decidualization and tail vein injection were performed following standard procedures as previously described (35, 66, 67). All mice used in this study were maintained in a barrier facility at Washington University School of Medicine following the institution’s regulations with an approved protocol.

### Uterine stromal cell isolation and organ culture.

Uteri of 2.5 dpc mice were collected, rinsed in cold Hank’s Balanced Salt Solution (HBSS; Gibco), cut into 2 to 3 mm pieces, and digested in 1% trypsin (MilliporeSigma) in HBSS for 1 hour at room temperature with gentle shaking. After incubation, LE of each uterine segment was gently squeezed out using fine forceps along the longitudinal axis of the uterus. The remaining uterine tissues were transferred to a fresh tube, further digested in 0.25% trypsin and 1 mg/mL collagenase (MilliporeSigma) in HBSS for 30 minutes at 37°C with gentle shaking, and dissociated by pipetting several times after incubation. Cell suspension and tissue remnants were filtered through a 70 μm nylon filter, and stromal cells were resuspended in hESC culture media (phenol red–free DMEM/F12, 7.5% charcoal-stripped FBS, 1× nonessential amino acids, 1× antibiotic-antimycotic). Stromal cells were seeded in 12-well plates, and follistatin (Sino Biological US Inc) was added to the culture media at indicated concentrations. For organ culture, 2.5 dpc uteri were cut into 2 to 3 mm segments and placed on the membrane of multiwall inserts. The inserts were then placed into 12-well culture plates containing 0.5 mL culture medium, and 10 agarose beads soaked in 100 ng/μL follistatin (Sino Biological US Inc) or BSA were transferred into the lumen of each segment (68). Both primary stromal cells and uterine organ cultures were harvested 48 hours after for RNA isolation.

### hESC culture.

Immortalized hESCs were previously characterized (69) and purchased from the American Type Culture Collection (CRL-4003). hESCs were maintained in hESC culture media plus 1× Insulin/Transferrin/Selenium (Gibco) in a humidified 37°C incubator supplied with 5% CO_2_. To induce decidualization in vitro, culture medium was replaced with induction medium (hESC medium plus 36 nM 17β-estradiol, 1 μM medroxyprogesterone/MPA, 0.1 mM cAMP), and hESCs were allowed to decidualize for 96 hours before RNA extraction. Gene knockdown experiments were performed using DharmaFECT4 transfection reagent (GE Healthcare Dharmacon) and Silencer Select Validated siRNAs (Thermo Fisher Scientific, catalog 4427038, *RARA*, siRNA s11801; *RARB*, siRNA s534565; *RARG*, siRNA s11807) following the manufacturer’s instructions. The morning after transfection, 10× induction cocktail topper was added to the culture to a final concentration of 1×, and cells were cultured for an additional 72 hours before harvest. For RAR antagonist treatment, hESCs were treated with induction medium with or without AGN194310 (MilliporeSigma) at indicated concentrations for 72 hours, before harvest for RNA extraction.

### RNA isolation and real-time RT-PCR.

RNA was extracted in RNA STAT-60 reagent following the manufacturer’s instructions (Tel Test Inc). Reverse transcription was performed using the High Capacity cDNA Reverse Transcription kit (Applied Biosystems), and qPCR was performed on ViiA 7 Real-Time PCR System (Applied Biosystems) using PowerUp SYBR Green Master Mix (Applied Biosystems). All results were repeated in 3 biological replicates unless specified and relative gene expression changes were determined by ΔΔ Ct method (normalized to housekeeping gene, *Rpl7*). Primers are listed in Supplemental Table 2.

### Histology, immunofluorescence, and alkaline phosphatase activity assay.

Tissues fixed in Bouin’s fixative were processed for serial dehydration and embedding at the Developmental Biology Histology Core at Washington University School of Medicine. Eight-micrometer paraffin sections were used for H&E staining and immunofluorescence (70). All antibodies were used at 1:1000 dilution in blocking solution (1% BSA, 3% normal goat serum in PBS): CDH1 (BD Biosciences), phospho-histone H3 (MilliporeSigma), Alexa Fluor 594 goat anti-rabbit, and Alexa Fluor 488 goat anti-mouse (Life Technologies). Paraformaldehyde-fixed paraffin-embedded sections were used for alkaline phosphatase (AP) activity assay. The sections were dewaxed, rehydrated, and washed in PBS. Rehydrated sections were subsequently incubated in freshly prepared AP staining solution containing 0.33 mg/mL nitro blue tetrazolium (Roche) and 0.165 mg/mL 5-bromo-4-chloro-3-indolyl-phosphate (Roche) in AP buffer (100 mM Tris-Cl, pH 9.0, 150 mM NaCl, 1 mM MgCl_2_) for color development. Antibody catalog numbers are listed in Supplemental Table 3.

### Ovarian hormone analyses.

Whole blood was collected by cardiac puncture and allowed to coagulate in 1.5 mL Eppendorf tubes at room temperature for 20 minutes. The blood samples were centrifuged at 800*g* for 10 minutes at 4°C, and supernatant (serum) aliquoted and stored at –80°C until use. Cayman Chemical ELISA kits for detection of E2 (501890) and P4 (582601) were used to determine serum hormone levels following the manufacturer’s instructions. Plates were read on a Bio-Rad 3550 microplate reader at the wavelength of 405 nm, and data were processed in Microsoft Excel and visualized in GraphPad Prism. Four biological replicates were tested and presented for each genotype.

### In situ hybridization.

In situ hybridization was performed on PFA-fixed, paraffin-embedded, 8 μm tissue sections using RNAscope 2.5 HD Assay-RED kit (Advanced Cell Diagnostics). Gene-specific double-“Z” oligo probes compatible with the kit were ordered from Advanced Cell Diagnostics (probe-Mm-*Bmp2*, catalog 406661; probe-Mm-*Fst*, catalog 454331), and detailed in situ procedure has been previously described (35).

### Statistics.

All experimental groups contained 3 biological replicates, if not specified otherwise. Two-tailed Student’s *t* test assuming unequal variance was performed to compare means of the experimental groups. For dose response experiments, 1-way ANOVA with post hoc Tukey’s honestly significant difference test was performed. Data are presented as mean ± SD, with raw individual experimental data displayed as dot plot overlay, and a *P* value of less than 0.05 was considered statistically significant.

### Study approval.

The animal studies included herein were reviewed and approved by the IACUC of Washington University. All studies were performed to the current standards of the American Association for Laboratory Animal Science so as to minimize pain, suffering, and total animals necessary for conclusive findings.

## Author contributions

YY, MEH, and LM designed the study. YY, MEH, and SBC conducted experiments. YY, MEH, SBC, RK, and LM analyzed data and wrote the manuscript.

## Supplementary Material

Supplemental data

## Figures and Tables

**Table 1:**
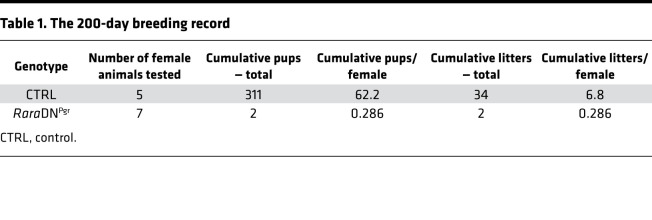
The 200-day breeding record

## Version 1 07/22/2021

In-Press Preview

## Version 2 09/08/2021

Electronic publication
