# Supplementary material for: Signaling through retinoic acid receptors is essential for mammalian uterine receptivity and decidualization
Source: JCI Insight. 2021 Sep 8;6(17):e150254. doi: 10.1172/jci.insight.150254 (PMC8492326; doi:10.1172/jci.insight.150254)
Supplement: Supplemental data [file jciinsight-6-150254-s090.pdf]

Supplemental Figures

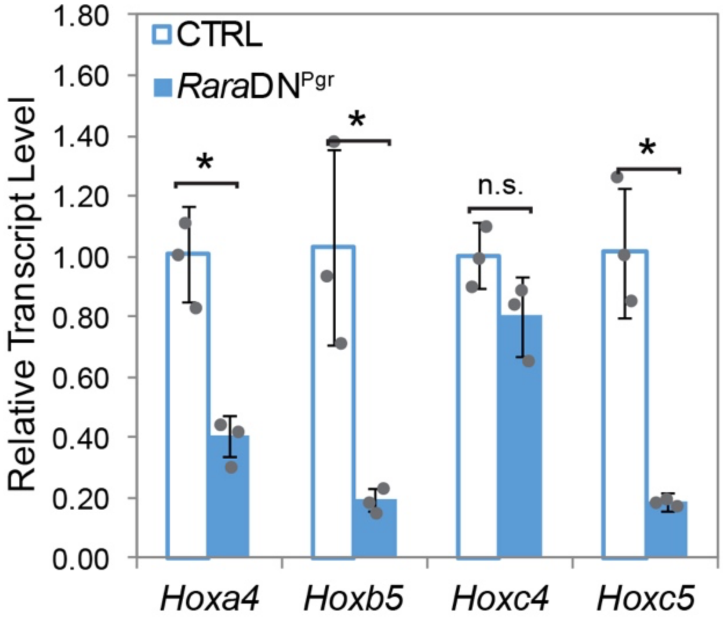

Fig. S1. Expression of known RAR transcriptional targets in unstimulated uterine tissue of ovariectomized CTRL and *Rara*DN^{Pgr} females. Asterisks indicate p <0.05.

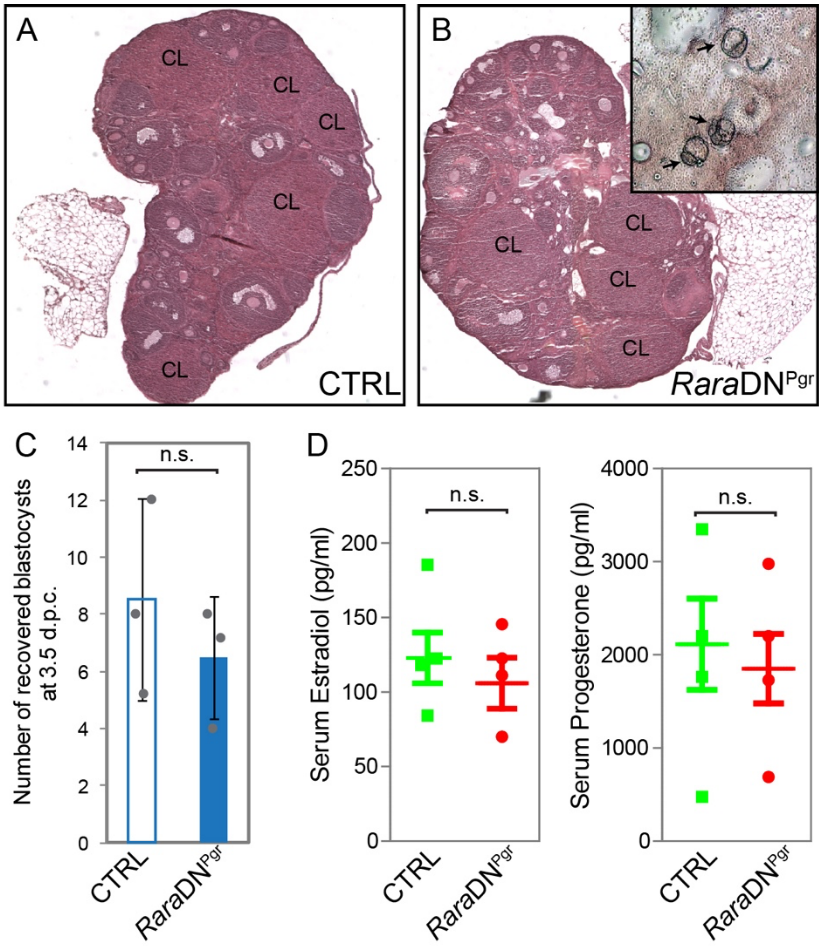

Fig. S2. Ovarian morphology and function appear normal in *Rara*DN^{Pgr} females. (A, B) H&E staining of 3.5 dpc ovaries of *Rara*DN^{Pgr} and CTRL mice. CL, corpus luteum; inset in B is a bright field image of blastocysts (arrows) flushed from *Rara*DN^{Pgr} oviducts and uterine horns at 3.5 dpc. (C) Quantification of recovered blastocysts from oviduct/uterine flush at 3.5 dpc (CTRL, 8.3 ± 3.5, n=3; *Rara*DN^{Pgr}, 6.3 ± 2.1, n=3; p=0.27). (D) Serum level of E2 (CTRL, 122.5 ± 19.4 pg/ml , n=4; *Rara*DN^{Pgr}, 104.0 ± 20.9 pg/ml, n=4; p=0.82) and P4 (CTRL, 2201.9 ± 498.9 pg/ml, n=4; *Rara*DN^{Pgr}, 1764.6 ± 414.4 pg/ml , n=4; p=0.71) at 3.5 dpc determined by ELISA. n.s. not significant.

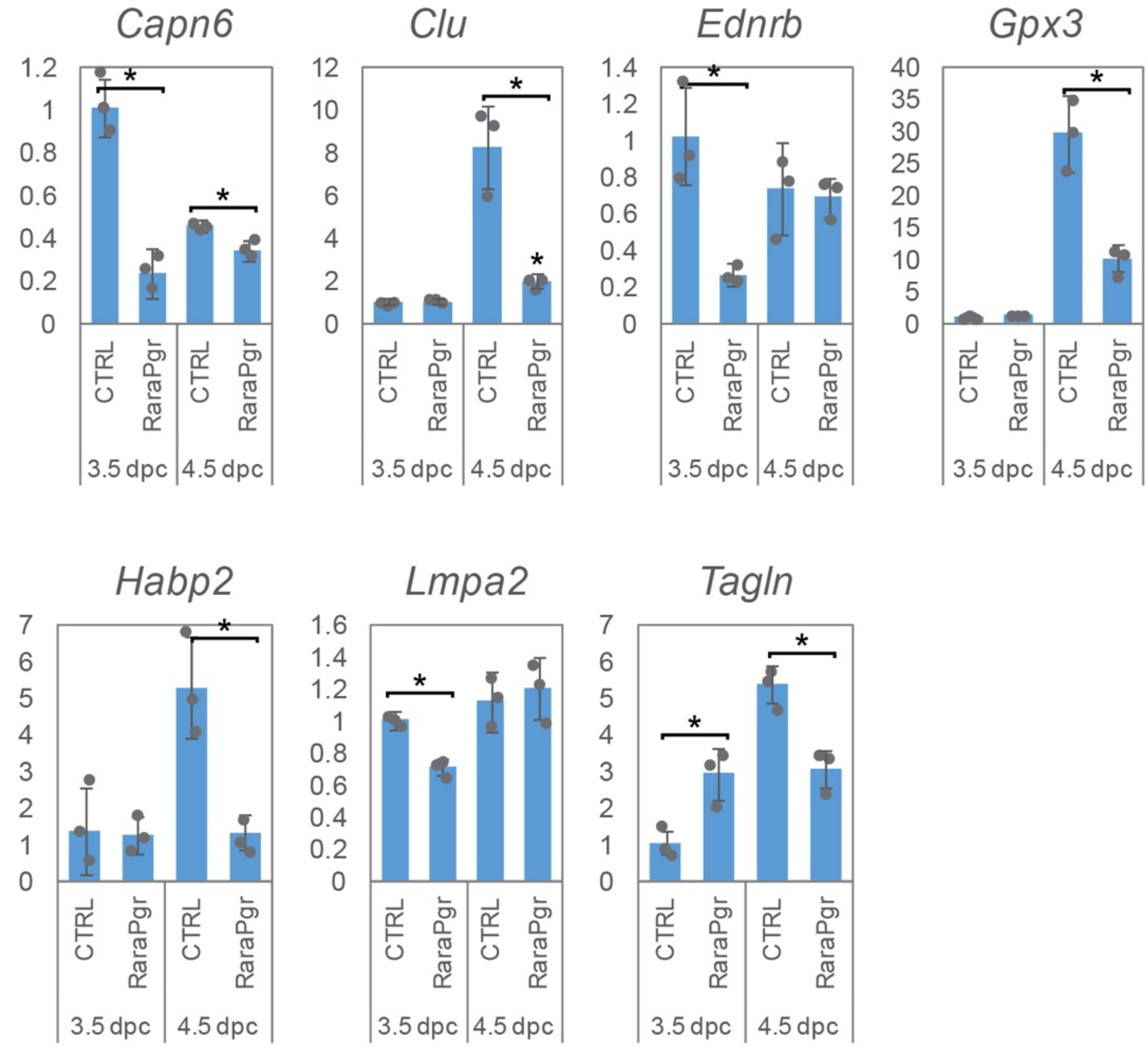

Fig. S3. Expression of gold standard receptivity biomarkers in the CTRL and *Rara*DN^{Pgr} females during peri-implantation. Asterisks indicate *p* <0.05.

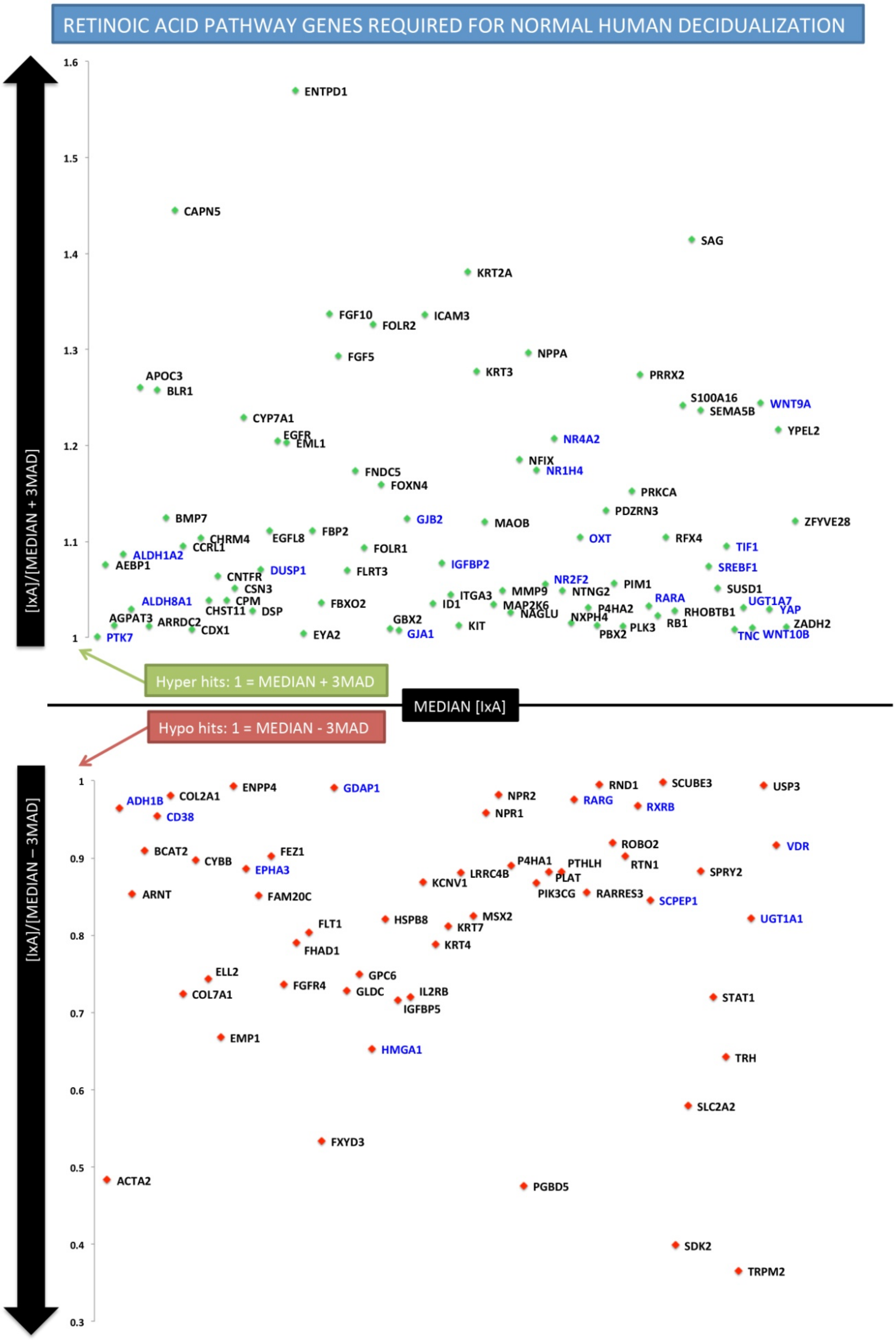
RETINOIC ACID PATHWAY GENES REQUIRED FOR NORMAL HUMAN DECIDUALIZATION
[IxA]/[MEDIAN + 3MAD]
1.6
1.5
1.4
1.3
1.2
1.1
1
ENTPD1
CAPN5
SAG
KRT2A
FGF10
ICAM3
FOLR2
NPPA
FGF5
KRT3
PRRX2
APOC3
BLR1
S100A16
SEMA5B
WNT9A
CYP7A1
YPEL2
EGFR
EML1
NR4A2
NFIX
FNDC5
NR1H4
FOXN4
PRKCA
PDZRN3
BMP7
GJB2
MAOB
ZFYVE28
FBP2
CHRM4
EGFL8
OXT
RFX4
CCRL1
FOLR1
TIF1
ALDH1A2
IGFBP2
AEBP1
SREBF1
DUSP1
FLRT3
CNTFR
NR2F2
PIM1
CSN3
MMP9
NTNG2
SUSD1
ITGA3
RARA
ALDH8A1
CPM
FBXO2
ID1
MAP2K6
P4HA2
UGT1A7
CHST11
DSP
NAGLU
RHOBTB1
YAP
AGPAT3
GBX2
NXPH4
RB1
ZADH2
ARRDC2
KIT
PLK3
CDX1
EYA2
GJA1
PBX2
TNC
WNT10B
PTK7
Hyper hits: 1 = MEDIAN + 3MAD
MEDIAN [IxA]
Hypo hits: 1 = MEDIAN - 3MAD
[IxA]/[MEDIAN – 3MAD]
1
0.9
0.8
0.7
0.6
0.5
0.4
0.3
RND1
SCUBE3
USP3
ENPP4
GDAP1
ADH1B
COL2A1
NPR2
RARG
RXRB
CD38
NPR1
ROBO2
VDR
BCAT2
FEZ1
CYBB
P4HA1
RTN1
EPHA3
LRRC4B
PTHLH
SPRY2
KCNV1
PLAT
ARNT
FAM20C
PIK3CG
RARRES3
SCPEP1
MSX2
FLT1
HSPB8
KRT7
UGT1A1
FHAD1
KRT4
ELL2
GPC6
FGFR4
GLDC
IL2RB
COL7A1
IGFBP5
STAT1
EMP1
HMGA1
TRH
SLC2A2
FXYD3
ACTA2
PGBD5
SDK2
TRPM2

21

Fig. S4. Genes for which silencing by siRNA affects hESC decidualization in response to ovarian hormones were cross referenced to retinoic acid (RA) responsive genes shown in black text, as well as retinoic acid upstream pathway genes (identified by searching amigo.geneontology.org for term "retinoic acid" and filtering for human genes) shown in blue text. Eighty-two RA genes (19 upstream and 63 targets) for which silencing results in hyper-responsiveness of hESCs to ovarian hormones are indicated by green dots and normalized to $(IxA)_{MEDIAN+3MAD}$. Fifty-four RA genes (10 upstream and 44 targets) for which silencing results in hypo-responsiveness of hESCs to ovarian hormones are indicated by red dots and normalized to $(IxA)_{MEDIAN-3MAD}$.

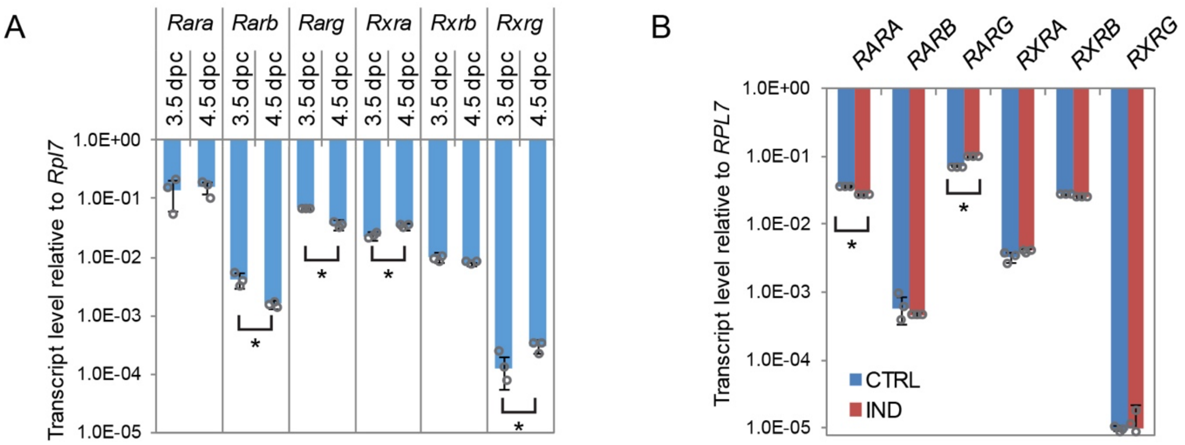

Fig. S5. (A) Expression of endogenous *Rar* and *Rxr* genes in wild-type mouse uterus during peri-implantation period relative to housekeeping gene, *Rpl7* (1 means ΔCt=0). (B) Expression of *RAR* and *RXR* genes in cultured hESCs decidualized in vitro relative to *RPL7* (CTRL, control medium; IND, induction medium). Asterisks indicate $p < 0.05$.

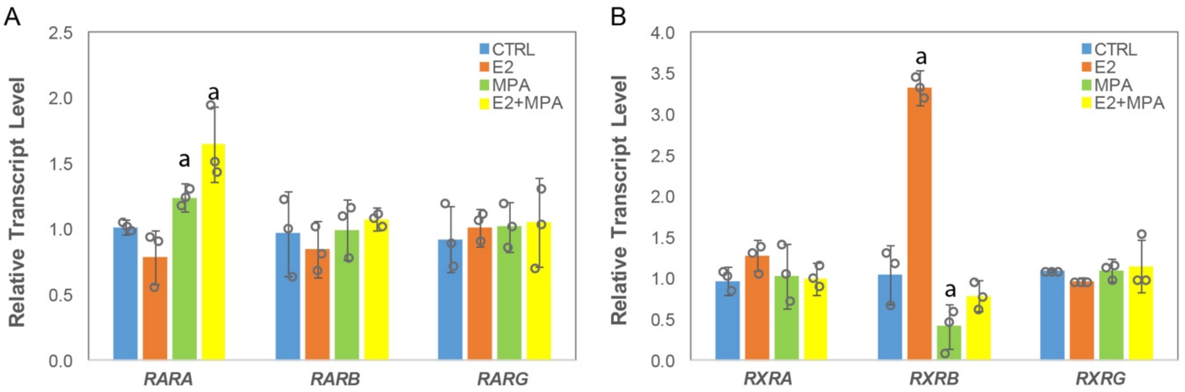

Fig. S6. Hormonal regulation of *RAR* (A) and *RXR* genes (B) in cultured hESCs exposed to E2, MPA or combination of the two for 24 hours. a indicates p <0.05 by one-way ANOVA between drug group and control group.

Table S1. Vitamin A content in the top 25 best-selling prenatal vitamins in the USA

Brand	Description	ASIN	amazon.com selling rank	percent daily value for pregnant and lactating persons	precursor type	raw units
Eu Natural	Conception Fertility Prenatal Vitamins – Regulate Your Cycle, Balance Hormones, Aid Ovulation – Myo-Inositol, Vitex, Folate Folic Acid Pills – 60 Vegetarian Soft Capsules	B013RMKCV4	1	none	none	none
SmartyPants	SmartyPants Prenatal Formula Daily Gummy Multivitamin: Vitamin C, D3, & Zinc for Immunity, Gluten Free, Folate, Omega 3 Fish Oil (DHA/EPA), 120 Count (30 Day Supply)	B00VFYYAC4	2	40	50% beta carotene and 50% retinyl palmitate USP	520 mcg
Solimo	Amazon Brand - Solimo Prenatal Vitamins & DHA - Pregnancy Wellness - 90 Gummies (45-Day Supply)	B07BBNRSBJ	3	92	retinyl acetate	1200 mcg
Vitafusion	(Family Bundle)	B003IP8BC8	4	50	retinyl palmitate	650 mcg RAE
Nature Made	Prenatal Multi + DHA Softgels, Prenatal Vitamins & Minerals for Baby's Development & Mom's Nutritional Support, Clinically Proven Absorption of Folic Acid and Iron, 110 Count	B07BXVFC32	5	59	beta carotene	770 mcg
Thorne Research	Thorne Research - Basic Prenatal - Folate Multivitamin for Pregnant and Lactating Women - 90 Capsule	B00O59SH3E	6	81	43% beta carotene and 57% as retinyl palmitate	1050 mcg
Garden of Life	Organics Prenatal Gummy Vitamins - Berry - Organic, Non-GMO, Vegan, Kosher Complete Multi - Methyl B12, D3 & Folate - Gluten, Soy & Dairy Free - 120 Real Fruit Chew Gummies	B06XSD99V3	7	35	not stated	450 mcg
Garden of Life	Garden of Life Vitamin Code Raw Prenatal Multivitamin, Whole Food Prenatal Vitamins with Iron, Folate not Folic Acid, Best Vegetarian Gluten Free Prenatals for Women, 90 Capsules	B0032EZOAM	8	100	beta carotene	1300 mcg
Garden of Life	Garden of Life mykind Organics Prenatal Vitamins - 30 Tablets, Prenatal Once Daily Whole Food Vitamins for Women with Folate not Folic Acid, Vitamin D3, Iron, Vegan One a Day Prenatal Multivitamin	B016O9HCHE	9	100	beta carotene	1300 mcg
Rainbow Light	Rainbow Light Prenatal One Vitamins + Superfoods, Non-GMO, Vegetarian & Gluten Free, Clinically Proven Absorption of Vitamin D, B2, B5, folate, calcium, iron, zinc, 90 Tablets (Package May Vary)	B000EE8036	10	100	beta carotene	1300 mcg
Pink Stork	Pink Stork Total Prenatal Vitamins with DHA and Folic Acid: Doctor Formulated, Folate + Iron + Biotin + Vitamin D + Vitamin C + Zinc, Women-Owned, 60 Vegetarian Capsules	B07G4C85XX	11	100	retinyl palmitate	1300 mcg RAE
MegaFood	MegaFood, Baby & Me 2, Prenatal and Postnatal Vitamin with Active Form of Folic Acid, Iron, Choline, Non-GMO, 60 Tablets	B01CQ3E4AK	12	58	beta carotene	750 mcg RAE
SmartyPants	Daily Organic Gummy Prenatal Multivitamin: Probiotic, Vitamin C, D3 & Zinc for Immunity, Biotin, Omega 3, Selenium, Methyl B12 for Energy by SmartyPants (120 Ct, 30 Day Supply)Packaging May Vary	B079YLCC3T	13	40	50% beta carotene and 50% retinyl palmitate USP	520 mcg
ACTIF	Actif Organic Prenatal Vitamin with 25+ Organic Vitamins, 100% Natural, DHA, EPA, Omega 3, and Organic Herbal Blend - Non-GMO, 90 Count,	B01LVYT5RL	14	100	beta carotene	5000 IU
Garden of Life	Garden of Life Prenatal Vitamins - mykind Organics Prenatal Multi - 90 Tablets, Vegan Whole Food Multivitamin, Folate not Folic Acid & Stomach Soothing Blend, Organic Prenatals Supplements for Women	B00K5NELM0	15	100	beta carotene	1300 mcg
New Chapter	New Chapter Perfect Prenatal Vitamins - 96ct, Organic Prenatal Vitamins, Non-GMO Ingredients for Healthy Baby & Mom - Folate (Methylfolate), Iron, Vitamin D3, Fermented with Whole Foods and Probiotic	B018OSDOJ8	16	100	beta carotene	1300 mcg
ONE A DAY	One A Day Women's Prenatal Advanced Complete Multivitamin with Brain Support* with Choline, Folic Acid, Omega-3 DHA & Iron for Pre, During and Post Pregnancy, 60+60 Count (120 Count Total Set)	B084PHB9WZ	17	50	beta carotene	650 mcg
Concieve Plus	Conceive Plus Fertility Prenatal Vitamins Women – Regulate Ovulation, Monthly Cycles, Balance Hormones - Myo-Inositol, Folate Folic Acid Pills - 60 Vegetarian Soft Capsules	B0734WQ6QY	18	none	none	none
Best Nest Wellnes	Mama Bird AM PM Prenatal Multi+, Methylfolate (Folic Acid), Methylcobalamin, Choline, Natural Whole Food Organic Herbal Blend, Vegan, Twice Daily Vitamin, Immune Support, 60 Ct, Best Nest Wellnes	B075X1JCKY	19	92	50% beta carotene and 50% retinyl palmitate USP	1200 mcg RAE
Nature Made	Nature Made Prenatal + DHA 200 mg Softgels (.150 Count)	B07KVMMMCZ	20	59	beta carotene	770 mcg
Zahler	Zahler Prenatal DHA, Premium Prenatal Vitamins for Mother and Child, New and Improved Prenatal with DHA! Certified Kosher, 60 Count	B01KYBTSQQ	21	50	50% beta carotene and 50% retinyl palmitate USP	650 mcg RAE
Rainbow Light	Rainbow Light Prenatal Daily Duo: Prenatal One Multivitamin & Prenatal Dhal, 30 Tablets & 30 Softgels (Package May Vary)	B001HCDTCG	22	100	beta carotene	1300 mcg
Rainbow Light	Rainbow Light Prenatal Multivitamin, Immune Support, 120 Capsules (Package May Vary)	B0033ZDGQY	23	29	beta carotene	375 mcg
Pink Stork	Pink Stork Total Postnatal + DHA: Support for Postpartum + Breastfeeding Vitamins, Nutrients for Mom + Baby, Prenatal Vitamins for After Baby, Women-Owned, 60 Capsules	B07NQPBXLH	24	185	retinyl palmitate	2400 mcg RAE
Lunaki	Prenatal Vitamins Gummies for Women with Iron and Folic Acid, Chewable Multivitamin Gummy Without Corn Syrup 30 Day Supply	B08GGBSGH8	25	20	all-trans retinyl acetate	1000 IU

Table S2. Primer sequences used for qPCR

Sequence Name	Sequence
*hRARA F	TAC TGC CGA CTG CAG AAG TG
hRARA R	CGT CAG CGT GTA GCT CTC AG
hRARB F	GAA ACA GGC CTT CTC AGT GC
hRARB R	TTG CTG GGT CGT CTT TTT CT
hRXRA F	TGG ACG CCT TCT CCA TAG TC
hRXRA R	GAC ACT TTC TTC CCC ACC AA
hRXRB F	CCA TGA TTT GGG GTG ATT TC
hRXRB R	TCC CCA TTT CCA CTC TTC AG
hRXRG F	CGG GAT TGG AAA CAT GAA CT
hRXRG R	TAC CCC GTA GTG CTT TCC TG
hIGFBP1 F	TTG GGA CGC CAT CAG TAC CTA
hIGFBP1 R	TTG GCT AAA CTC TCT ACG ACT CT
hPRL F	TGA CCC TTC GAG ACC TGT TTG
hPRL R	CTT GCT CCT TGT CTT CGG G
hRPL7 F	CCA AAT TGG CGT TTG TCA TC
hRPL7 R	AAG ATT TGA CGA AGG CGA AG
mAreg F	GTC TTA GGC TCA GGC CAT TA
mAreg R	GCT TAT GGT GGA AAC CTC TC
mEgr1 F	AGA CGA GTT ATC CCA GCC AA
mEgr1 R	GGT CGG AGG ATT GGT CAT GC
mFoxo1 F	CCC AGG CCG GAG TTT AAC C
mFoxo1 R	GTT GCT CAT AAA GTC GGT GCT
mFst F	TGC TGC TAC TCT GCC AGT TC
mFst R	TGC TGC AAC ACT CTT CCT TG
mHoxa10 F	GAC CTG GCT GGT TGA AAA AG
mHoxa10 R	CCA GGC AAG CAA GAC CTT AG
mHsd11b1 F	CAG AAA TGC TCC AGG GAA AGA
mHsd11b1 R	GCA GTC AAT ACC ACA TGG GC
mIgfbp5 F	AGA CAG CAC AAC TTC AGT TC
mIgfbp5 R	CAC AGG GAA GGA TGT TTG AG
mIhh F	CTC TTG CCT ACA AGC AGT TCA
mIhh R	CCG TGT TCT CCT CGT CCT T
mEsr1 F	CCT CCC GCC TTC TAC AGG T
mEsr1 R	CAC ACG GCA CAG TAG CGA G
mEsr2 F	CTG TGC CTC TTC TCA CAA GG
mEsr2 R	GCT CCA AGG GTA GGA TGG AC
mPgr F	CTC CGG GAC CGA ACA GAG T
mPgr R	ACA ACA ACC CTT TGG TAG CAG
mGata2 F	CAC CCC GCC GTA TTG AAT G
mGata2 R	CCT GCG AGT CGA GAT GGT TG
mHand2 F	CAG GAC TCA GAG CAT CAA CA
mHand2 R	AGG TAG GCG ATG TAT CTG GTG
mHbegf F	CGG GGA GTG CAG ATA CCT G
mHbegf R	TTC TCC ACT GGT AGA GTC AGC
mLpar3 F	ACC ATG CGG AAG ATG ATC TG
mLpar3 R	AGC CGT TTT TAT TGC ACA CC
mRara F	GAC CAG ATC ACC CTC CTC AA
mRara R	TCA GGG TCA GTC CAT CTG AG
mRarb F	CCA GAT CAC CCT GCT CAA AG
mRarb R	GCA TTG TGC ATC TGA GTT CG
mRarg F	AGG TCA CCA GAA ATC GAT GC
mRarg R	CGA GCC CTC CTC TTT TAC CT
mRxra F	GTC GAG CCC AAG ACT GAG AC
mRxra R	AGA GCT GCT TGT CTG CTG CT
mRxrb F	CCC TTC CCA GTC ATC AGT TC

mRxrb R	GAG CGA CAC TGT GGA GTT GA
mRxrg F	CCT ACG GTG ACA TGA ACG TG
mRxrg R	GAA TGA CCT GGT CCT CCA AG
mCdx1 F	ACG CCT AGA GCT GGA AAA GG
mCdx1 R	GTT TAC TTT GCG CTC CTT GG
mGbx2 F	AGA CGG CAA AGC CTT CTT G
mGbx2 R	CGG GTC ATC TTC CAC CTT T
mMsx1 F	AGC CCC GAG AAA CTA GAT CG
mMsx1 R	TTG GTC TTG TGC TTG CGT AG
mMsx2 F	AAT TCC GAA GAC GGA GCA C
mMsx2 R	CGG TTG GTC TTG TGT TTC CT
mSox17 F	GAT GCG GGA TAC GCC AGT G
mSox17 R	CCA CCT CGC CTT TCA CCT TTA
mFgfr3 F	GGA GGA CGT GGC TGA AGA C
mFgfr3 R	GGA GCT TGA TGC CCC CAA T
mMmp9 F	CAA TCC TTG CAA TGT GGA TG
mMmp9 R	AGT AAG GAA GGG GCC CTG TA
mPrrx2 F	CGT GGC ACC AAA CGA AAG AAG
mPrrx2 R	GTA GTG TGT GCG CTC AAA TAC A
mActa2 F	GTC CCA GAC ATC AGG GAG TA
mActa2 R	TCG GAT ACT TCA GCG TCA GG
mEml1 F	ACT TGG ACT GGT CCG TGA AC
mEml1 R	TCC CAA GGT GCA GGT ATA GG
mFxyd3 F	ATT TGT GCA GGG ATT CTC TGT G
mFxyd3 R	GCC TGG TGT GAT GAG AGG TG
mGbx2 F	AGA CGG CAA AGC CTT CTT G
mGbx2 R	CGG GTC ATC TTC CAC CTT T
mRtn1 F	CTG TGG CTA GGC CTT TCT TG
mRtn1 R	TGT GTG TGG GGA AAT GCT AA
mTrh F	AGG GAC CTT GGC TGA TGA TG
mTrh R	CCG GAC CTG GAC TTT CTC C
mCapn6 F	GGA AGC GTC CAC AGG ACA T
mCapn6 R	GTG GTT GGG AAT TGC CTT TG
mEdnrb F	GTG GCT TCT TGG GGG TAT GG
mEdnrb R	TCT TAG TGG GTG GCG TCA TT
mGpx3 F	CCT TTT AAG CAG TAT GCA GG
mGpx3 R	CAA GCC AAA TGG CCC AAG TT

*m and h at the start of primer name stands for mouse and human, respectively; F and R at the end of primer stands for forward primer and reverse primer, respectively.

Table S3. List of commercial antibodies used in the study.

Antibody	Vendor	Catalog Number
pHH3	Millipore	06-570
Ecad	BD	610181
ER	Santa Cruz	sc-542
PR	Santa Cruz	sc-7208
HAND2	Santa Cruz	sc-398167
pSMAD1/5/8	Cell Signalling	#9511
GAPDH	Cell Signalling	#5174
